# Evaluation of the effectiveness of the modified nutrition risk in the critically ill (mNUTRIC) score in critically ill patients affected by COVID-19 admitted to the intensive care unit (ICU)

**DOI:** 10.1186/s40795-022-00659-9

**Published:** 2022-12-30

**Authors:** Babak Alikiaii, Seyed Taghi Hashemi, Zahra Kiani, Zahra Heidari, Behzad Nazemroaya, Mohammad Golparvar, Somayyeh Daneshmanesh, Shirin Fattahpour, Sepide Amini, Mehrnaz Shojaei, Mohammad Bagherniya

**Affiliations:** 1 Anesthesia and Critical Care Research Center, Isfahan University of Medical Sciences, Isfahan, Iran; 2 Department of Community Nutrition, Student Research Committee, School of Nutrition and Food Science, Isfahan University of Medical Sciences, Isfahan, Iran; 3 Department of Biostatistics and Epidemiology, School of Health, Isfahan University of Medical Sciences, Isfahan, Iran; 4 Isfahan Cardiac Rehabilitation Research Center, Cardiovascular Research Institute, Isfahan University of Medical Sciences, Isfahan, Iran; 5 Craniofacial and Cleft Research Center, Isfahan University of Medical Sciences, Isfahan, Iran; 6 Nutrition and Food Security Research Center and Department of Community Nutrition, School of Nutrition and Food Science, Isfahan University of Medical Sciences, Isfahan, Iran

**Keywords:** NUTRIC score, ICU, COVID-19, Morbidity, Mortality

## Abstract

**Background:**

COVID-19 is a very harmful pandemic, and its recovery process is highly influenced by nutritional status; however, an appropriate nutritional scale has not yet been proposed for these patients. Therefore, the purpose of this study was to evaluate the effectiveness of the modified Nutrition Risk in the Critically ill (mNUTRIC) score in critically ill patients affected by COVID-19 admitted to the intensive care unit (ICU).

**Material and methods:**

This was a cross-sectional study performed on 204 critically ill patients affected by COVID-19 admitted to the ICU wards. Evaluated indicators include the mNUTRIC Score as well as demographic, and biochemical indicators.

**Results:**

A high percentage of COVID-19 patients (67.2%) had severe disease. Hospital and ICU stay (*p* > 0.001) and PH (*p* > 0.001) values were significantly lower in non-survivors than in survivors. mNUTRIC score (*p* > 0.001), PCO2 (*p* = 0.003), and CRP levels (*p* = 0.021) were significantly higher in non-survivors than survivors. mNUTRIC score had a direct correlation with age (*p* > 0.001), AST (*p* = 0.000), LDH (*p* = 0.026), and CRP (*p* = 0.014) and an inverse correlation with hospital duration (*p* = 0.031), albumin (*p* = 0.003) and PH (*p* < 0.001). Furthermore, there was a non-significant correlation between the mNUTRIC score and mortality chance (OR = 1.085, 95%CI [0.83, 1.42], *p* = 0.552). While, patients with more severe COVID-19 disease (OR = 8.057, 95%CI [1.33, 48.64], *p* = 0.023) and higher PCO2 (OR = 1.042, 95%CI [1.01, 1.08], *p* = 0.023) levels had higher odds of mortality.

**Conclusions:**

Our findings revealed that COVID-19 patients with higher CRP levels and lower PH had higher mortality and poor nutritional condition. Moreover, there was a non-significant association between the mNUTRIC score and mortality chance.

## Introduction

Today, COVID-19 has received a great deal of attention because of its very high prevalence and mortality [1, 2]. This disease, which is caused by a new virus called Severe acute respiratory syndrome coronavirus 2 (SARS-CoV-2) [3], can result in many complications, including pneumonia, coagulopathy, electrolyte imbalance, and can even lead to severe symptoms of acute respiratory distress syndrome (ARDS) and multiple organ failure [4, 5]. Also, this disease, in particular, has many effects on the immune system, including granulocyte and monocyte abnormalities and increased cytokines production [1, 6]. However, there is still no definitive and complete treatment protocol for the current coronavirus (COVID-19) [7]. Therefore, it is necessary to pay more attention to this disease [8].

COVID-19 is directly related to malnutrition, because generally, this disease can cause abnormal weight loss due to indigestion, loss of appetite, nausea, and dysphagia [4, 9–12]. Also, this disease, which is usually associated with severe infection, increases the patient’s need for energy by increasing the body’s metabolism. Therefore, the patient is eventually exposed to harmful malnutrition [9, 10, 13]. A recent meta-analysis found that the prevalence of malnutrition in COVID-19 patients was significantly high, which increased their risk of mortality because malnutrition could exacerbate their distressing symptoms [14]. Poor nutritional status is important because it affects the balance of immune function and thus increases systemic infection. Malnutrition also increases the length of hospital stay and the likelihood of recurrence [15, 16].

To reduce adverse clinical outcomes, the nutritional risk of critically ill patients should be identified as soon as possible [17]. Most nutrition screening tools are not specific to ICU patients [18], as they are rated in such a way that all critically ill patients are considered at high risk [19, 20]. However, not all critically ill patients have the same nutritional risk, as the study by Alberda et al. showed that increasing calorie or protein intake in these patients generally reduced mortality, but this effect was not the same in critically ill patients with different body mass indexes (BMIs) [17]. In their study, Heyland et al. presented the Nutrition Risk in the Critically ill (NUTRIC) score as a practical tool for critically ill patients, particularly for mechanically ventilated patients. This score assesses age, disease severity, and the number of days hospitalized before admission to the ICU [21]. Therefore, since malnutrition is common in COVID-19 patients and affects their recovery process [14], it is necessary to focus on the nutritional status of these patients with a useful nutrition screening tool. Thus, this study aimed to evaluate the effectiveness of the NUTRIC score as a screening system in critically ill patients affected by COVID-19 admitted to the ICU.

## Materials and methods

### Study design

In this cross-sectional study, the study sample was adult patients with COVID-19 who were admitted to the ICU of Al-Zahra Hospital in Isfahan from July to December 2021.

#### Inclusion criteria were as follow

1. Age > 18 years.

2. Definitive diagnosis of COVID-19 was based on reverse transcription-polymerase chain reaction (RT-PCR).

3. Critically ill patients admitted to ICU.

4. Intubated and ventilated patients.

5. Admitted to the ICU for more than 48 hours.

#### Exclusion criteria were as follows

1. Severe malnutrition: Weight loss> 5% in 1 month (> 15% in 3 months) or BMI < 18.5 plus impaired general condition or food intake 0–25% of normal in the past week.

2. Psychiatric disorders that led to severe malnutrition.

3. Patients who were in coma and whose feeding history was unknown.

4. Treated with appetite suppressants.

5. Pregnancy or breastfeeding.

6. Patients who died before spending 24 hours in the ICU.

7. Resistant vomiting (vomiting that does not respond to plasil treatment within 48 hours).

### Data collection and variables

In the present study, the modified Nutrition Risk in the Critically ill (mNUTRIC) score was used to assess the nutritional status of critically ill patients with COVID-19 admitted to the ICU. The NUTRIC score is related to the identification of malnourished patients who do not receive enough protein and energy to reduce malnutrition-related mortality by correcting their nutritional status [21]. The NUTRIC score evaluates various parameters, including the number of comorbidities and Interleukin-6 (IL-6) [21]. In this nutrition scale, disease severity is also obtained by Acute Physiology and Chronic Health disease Classification System II (APACHE II) [22] and Sequential Organ Failure Assessment (SOFA) scores [23]. The number of days hospitalized before admission to the ICU and the age of the patients is the other items considered in the NUTRIC score (Table 1) [21].

**Table 1:** NUTRIC score variables

Variable	Range	Point
Age	< 50	0
	50 - < 75	1
	≥ 75	2
APACHE II	< 15	0
	15 - < 20	1
	20–28	2
	≥ 28	3
SOFA	< 6	0
	6 - < 10	1
	≥ 10	2
Number of Co-morbidities	0–1	0
	≥ 2	1
Days from hospital to ICU admission	0 - < 1	0
	≥ 1	1
IL-6	0 - < 400	0
	≥ 400	1

When IL-6 is available if the score is 0 to 5, the risk of malnutrition is low and if it is 6 to 10, the risk is high and requires aggressive nutrition therapy. Sometimes IL-6 is not available. In the study of Heyland et al., a significant effect of IL-6 on the c-index was not observed, so they stated that if the IL-6 was not available, the mNUTRIC could be used, which deleted IL-6 [21]. Also, Rahman et al. observed that the absence of IL-6 had no significant effect on the NUTRIC score [24]. In this case, if the overall score is 0 to 4, the risk of malnutrition is low and if it is 5 to 9, the risk is high.

For included the patients in the study, on the third day after the patients were admitted to the ICU, if their conditions match the inclusion and exclusion criteria, we entered them into the study after obtaining informed written consent. Then, age, APACHE II, SOFA, number of co-morbidities, and days from hospital to ICU admission were evaluated according to the NUTRIC score. Cases of comorbidity included hypertension, diabetes mellitus, chronic renal failure, neurological disease, coronary artery disease, liver disease, chronic obstructive airway disease, and malignancy. Demographic information was also recorded. BMI was obtained by assessing patients’ weight with a Seca scale with an accuracy of 0.1 kg and height with an inelastic meter with an accuracy of 0.1 cm. However, if it was not possible for the patient to stand on the scales, the patient’s weight and height, which were measured in the hospital in the previous week, were recorded. If this option was also not possible, patients’ height and usual body weight were acquired by asking themselves or their guardians.

Mortality and morbidity were assessed from the beginning of the study until 14 days after discharge from the ICU. Arterial O2 pressure (PaO2) and CO2 pressure (PCO2) were assessed by breathing in normal room air.

### Assessment of the severity of the disease

In the present study, PaO2 / FiO2 was measured to classify patients based on the severity of acute respiratory distress syndrome (ARDS). Accordingly, PaO2 / FiO2 > 200 indicates mild, PaO2 / FiO2 101–200 indicates moderate and PaO2 / FiO2 ≤ 100 indicates severe ARDS [25].

### Laboratory measurements

To evaluate serum albumin, alanine aminotransferase (ALT), aspartate aminotransferase (AST), bilirubin (Total and Deconjugate), lactate dehydrogenase (LDH), creatine phosphokinase (CPK), reactive protein C (CRP), and blood PH, 5 ml of blood was obtained after 6 hours of fasting in the morning. To prevent spoilage of the sample, after centrifuging at room temperature for 10 minutes to isolate the serum, the serum was kept at − 80 °C. These blood factors were assessed by commercial kits (Pars Azmun, Karaj, Iran).

### Statistical analysis

We used SPSS software version 21 (IBM Corp., Armonk, NY, USA) for data analysis. We reported continuous data as mean and standard deviation (SD) or median (IQR) and categorical data as frequency and percentage and considered *P*-value < 0.05 as significant. Kolmogorov-Smirnov test, Q-Q plot, and skewness statistics were used to evaluate the normality of the data. Then, if the variables were abnormal, we used the logarithmic transformation approach. We used chi-square or independent t-test to examine the distribution of patients among the mortality status. This distribution was evaluated based on demographic characteristics, anthropometric and biochemical indicators, and the severity of COVID-19 disease. Correlation analysis was used to evaluate the relationship between the studied variables with the NUTRIC Score. Comparison of continuous variables with the NUTRIC Score was performed by analysis of variance (ANOVA) and the relationship between different parameters and death status was analyzed by binary logistic regression analysis.

## Results

**Fig. 1:**
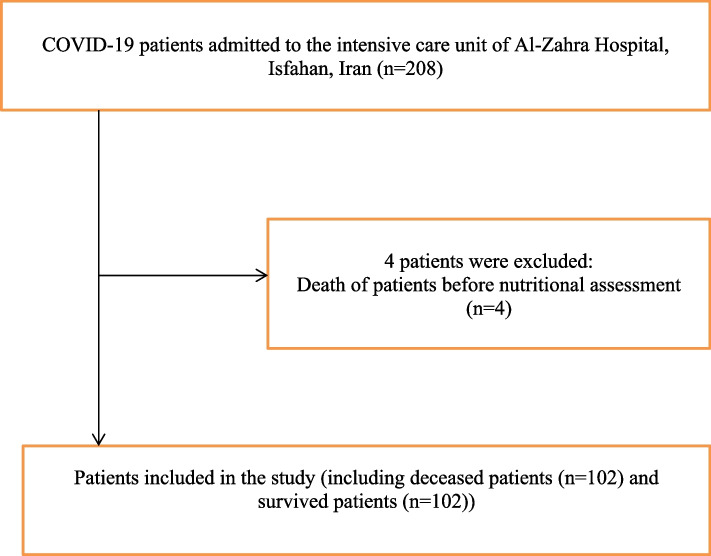
Patients process flowchart

From 208 patients who were admitted into the ICU, 4 patients died within 48 hours of admission (before evaluation of nutritional assessment), thus, were excluded from the analysis. Finally, 204 participants were included in this study. Out of these, 102 patients died and 102 patients survived (Fig. 1). Baseline characteristics of participants are shown in Table 2. The average age of all enrolled patients (*n* = 204) was 61.98 ± 15.27. The mean age was 60.34 ± 16.28 and 63.62 ± 14.07 in survivors and deceased patients respectively, (*p* = 0.125). Overall, 121 (59.3%) subjects were female and 83 (40.7%) were male. There was no significant difference between two groups of deceased patients and survivors in terms of sex (*p* = 0.318). The average length of hospital and ICU stay was 14.99 ± 9.31 and 11.04 ± 7.93 days, respectively in the total sample. The duration of both hospital and ICU stay was longer in survivors (17.76 ± 9.36 and 13.81 ± 7.54) than in deceased patients (12.21 ± 8.43 and 8.28 ± 7.36) (*p* < 0.001) (Table 2).

**Table 2:** Demographic characteristics, biochemical indices, nutritional status and Illness severity of the COVID-19 patients in intensive care unit

**Variable**			**All patients***N*=204	**Dead patients***N*=102	**Survivors***N*=102	*p*-value
Sex (frequency (percent))		Male	83 (40.7%)	45 (44.1%)	38 (37.3%)	0.318
		Female	121 (59.3%)	57 (55.9%)	64 (62.7%)	
Age (year)(Mean ± SD)			61.98 ± 15.27	63.62 ± 14.07	60.34 ± 16.28	0.125
APACHE2^a^			15.22±6.80	16.63±6.97	13.81±6.36	.003
SOFA^a^			5.65±2.96	6.42±3.07	4.89±2.64	<0.001
Duration of hospitalization (day)^a^			14.99 ± 9.31	12.21 ±8.43	17.76 ± 9.36	<0.001
Duration of hospitalization in ICU (day)^a^			11.04 ± 7.93	8.28 ± 7.36	13.81 ±7.54	<0.001
Comorbidities(frequency (percent))	No		75 (36.8%)	33 (32.4%)	42 (41.2%)	0.470
	Yes	Total	129 (63.2%)	69 (67.6%)	60 (58.8%)	
		Diabetes	21 (10.3%)	8 (7.8%)	13 (12.7%)	
		Hypertension	19 (9.3%)	10 (9.8%)	9 (8.8%)	
		Cancer	5 (2.5%)	3 (2.9%)	2 (2.0%)	
		Diabetes-hypertension	26 (12.7%)	12 (11.8%)	14 (13.7%)	
		IHD	20 (9.8%)	13 (12.7%)	7 (6.9%)	
		IHD-hypertension	9 (4.4%)	6 (5.9%)	3 (2.9%)	
		Renal	14 (6.9%)	10 (9.8%)	4 (3.9%)	
		Brain	5 (2.5%)	3 (2.9%)	2 (2.0%)	
		Others	10 (4.9%)	4 (3.9%)	6 (5.9%)	
NUTRIC Score^a^			4.08±1.58	4.46±1.38	3.71±1.68	.001
Albumin (g/dl)^a^			3.38±0.47	3.40±0.49	3.36±0.45	0.527
ALT (IU/L)^a^			58.38± 85.20	52.14+43.64	64.62± 112.28	0.297
AST (IU/L)^a^			68.81 ± 80.39	71.81± 86.50	65.80± 90.98	0.595
Bilirubin total (mg/dl)^a^			0.86± 0.85	0.81± 0.46	0.92± 1.11	.391
Bilirubin direct (mg/dl)^a^			0.31± 0.42	0.27± 0.14	0.34± 0.57	.276
LDH (U/L)^a^			966.32± 462.11	973.33± 446.45	959.31± 479.35	.829
CPK (U/L)^a^			376.56± 622.58	398.72± 569.93	354.41± 673.24	.612
CRP (mg/L)^a^			75.55± 37.23	81.56± 39.31	69.53± 34.18	.021
PaO2 (mmHg)^a^			53.32± 27.90	51.61± 27.77	55.03± 28.06	.382
INR^a^			1.29 ± 0.44	1.34±0.59	1.25±0.19	.114
PH^a^			7.34±0.10	7.31±0.10	7.36±0.08	<0.001
PCO2 (mmHg)^a^			41.20±11.95	43.68±14.15	38.72±8.64	.003
Illness severity(frequency(percent))^b^		mild	13 (6.4%)	2 (2.0%)	11 (10.8%)	.021
		Moderate	54 (26.5%)	25 (24.5%)	29 (28.4%)	
		Severe	137 (67.2%)	75 (73.5%)	62 (60.8%)	

Abbreviations: *ALT*Alanine aminotransferase, *AST*Aspartate aminotransferase, *LDH*Lactate dehydrogenase, *CPK*Creatinine phosphokinase, *CRP*Creactive protein, *PaO2*Partial pressure of oxygen, *IHD*Ischemic heart disease, *INR*International normalized ratio, *PCO2*Partial pressure of carbon dioxide, *APACHE2*Acute Physiology and Chronic Health Evaluation 2, *SOFA*Sequential Organ Failure Assessment, *NUTRIC*Nutrition Risk in the Critically ill, *ICU*Intensive care unit

^a^ Mean (SD); *p*-values were calculated based on independent sample t-test or Chi-square test. ^b^ PaO2 / FiO2> 200 indicates mild, PaO2 / FiO2 101-200 indicates moderate and PaO2 / FiO2 ≤ 100 indicates severe ARDS

Regarding laboratory findings, in all patients, the obtained mean of laboratory and the other parameters were as follow: ALT (58.38 ± 85.20 IU/L), AST (68.81 ± 80.39 IU/L), bilirubin total (0.86 ± 0.85 mg/dl), bilirubin direct (0.312 ± 0.42 mg/dl), LDH (966.32 ± 462.11 U/L), CPK (376.56 ± 622.58 U/L), CRP (75.55 ± 37.23 mg/dl), PaO2 (53.32 ± 27.90 mmHg), albumin (3.38 ± 0.47 g/Dl), international normalized ratio (INR) (1.29 ± 0.44), PH (7.34 ± 0.100), and PCO2 (41.20 ± 11.95 mmHg). A comparison of obtained values in two groups of survivors and deceased individuals revealed that there was a significant difference in CRP (*p* = 0.021), PH (*p* < 0.001), and PCO2 (*p* = 0.003) values (Table 2).

The mean APACHE II and SOFA scores in all participants were 15.22 ± 6.80 and 5.65 ± 2.96; both APACHE II (*p* = 0.003) and SOFA (*p* < 0.001) scores were significantly higher in deceased patients in comparison to the survivors. The average of mNUTRIC score in the total sample size was 4.08 ± 1.58. The mean of this score in deceased patients (4.46 ± 1.38) was significantly higher than in survivors (3.71 ± 1.68) (*p* = 0.001) (Table 2).

As shown in Table 2, using the following criteria, PaO2 / FiO2 > 200 indicates mild, PaO2 / FiO2 101–200 indicates moderate, and PaO2 / FiO2 ≤ 100 indicates severe ARDS, in total, 6.4% of individuals had mild, 26.5% had moderate and 67.2% had severe disease. The number of patients with mild and moderate diseases was higher in survivors, while the number of patients with severe diseases was higher in the deceased ones (*p* = 0.021). Overall, 75 (36.8%) patients had no comorbid disease. Among others, diabetes and hypertension together (12.7%) and diabetes (10.3%) were the most common comorbidities. *P*-value showed no significant difference between the two groups regarding comorbid diseases (*p* = 0.470) (Table 2).

We also analyzed the correlation of different parameters with the mNUTRIC score. It was found that there was a significant direct correlation between age (*p* < 0.001), AST (p < 0.001), LDH (*p* = 0.026), CRP (*p* = 0.014), APACHE II (p < 0.001), and SOFA (*p* = 0.002) with the NUTRIC score. Moreover, the mNUTRIC score was inversely correlated with hospital duration (*p* = 0.031), albumin (*p* = 0.003), and PH (p < 0.001). As presented in Table 3, there was no significant correlation between the mNUTRIC score and other indexes.

**Table 3:** The mean of patients’ demographic, anthropometrics and laboratory findings and the correlation of these parameters with modified Nutrition Risk in the critically ill score (mNUTRIC score)

Parameter	Mean ± SD	Correlation with NUTRIC	***p***-value
Age (year)	61.98 ± 15.27	0.321^b^	< 0.001
Duration of hospitalization (day)	14.99 ± 9.31	-0.151^a^	0.031
Duration of hospitalization in ICU (day)	11.04 ± 7.93	−0.119	0.089
ALT (IU/L)	58.38 ± 85.20	0.121	0.086
AST (IU/L)	68.81 ± 80.39	0.253^b^	< 0.001
Bilirubin total (mg/dl)	0.86 ± 0.85	−0.010	0.887
Bilirubin direct (mg/dl)	0.31 ± 0.42	0.007	0.916
LDH (U/L)	966.32 ± 462.11	0.156^a^	0.026
CPK (U/L)	376.56 ± 622.58	0.036	0.611
CRP (mg/L)	75.55 ± 37.23	0.172^a^	0.014
PaO2 (mmHg)	53.32 ± 27.90	−0.060	0.393
Albumin (g/dl)	3.38 ± 0.47	−0.206^b^	0.003
INR	1.29 ± 0.44	0.036	0.607
PH	7.34 ± 0.10	−0.363^b^	0.000
PCO2	41.20 ± 11.95	0.118	0.093
APACHE2	15.22 ± 6.80	0.434^b^	< 0.001
SOFA	5.65 ± 2.96	0.221^b^	0.002

*Abbreviations*: *ALT* Alanine aminotransferase, *AST* Aspartate aminotransferase, *LDH* Lactate dehydrogenase, *CPK* Creatinine phosphokinase, *CRP* C reactive protein, *PaO2* Partial pressure of oxygen, *INR* International normalized ratio, *PCO2* Partial pressure of carbon dioxide, *APACHE2* Acute Physiology and Chronic Health Evaluation 2, *SOFA* Sequential Organ Failure Assessment, *NUTRIC* Nutrition Risk in the Critically ill, *ICU* Intensive care unit

^a^Correlation is significant at the 0.05 level (2-tailed)

^b^Correlation is significant at the 0.01 level (2-tailed)

According to the results of logistic regression analysis, patients with severe disease had higher mortality chance (OR = 8.057, 95%CI [1.33, 48.64], *p* = 0.023). Increasing PCO2 levels were also associated with higher mortality chance (OR = 1.042, 95%CI [1.01, 1.08], p = 0.023). There was no significant correlation between the mNUTRIC score and the chance of death in participants (OR = 1.085, 95%CI [0.83, 1.42], *p* = 0.552) (Table 4).

**Table 4:** The correlation between mNUTRIC score, disease severity, hospital and ICU duration, C-reactive protein, PH, partial pressure of carbon dioxide, Acute Physiology and Chronic Health Evaluation 2, Sequential Organ Failure Assessment and mortality of COVID-19 Patients

Variable	Odds ratio	95% confidence interval
[Severity of Diseases = 3]^a^ Severe	8.057	[1.33–48.64]
[Severity of Diseases = 2]^a^ Moderate	4.714	[0.84–26.35]
[Severity of Diseases = 1]^a^ Mild	1	–
Hospital duration	1.016	[0.91–1.12]
Duration of admission in the ICU	.885	[0.78–1.00]
CRP	0.996	[0.98–1.01]
PH	0.055	[0.00–3.25]
PCO2	1.042	[1.01–1.08]
APACHE2	1.034	[0.95–1.11]
SOFA	1.172	[0.99–1.38]
mNUTRIC Score	1.085	[0.83–1.42]

*Abbreviations*: *mNUTRIC score* modified Nutrition Risk in the critically ill score, *ICU* Intensive care unit, *CRP* C reactive protein, *PCO2* Partial pressure of carbon dioxide, *APACHE2* Acute Physiology and Chronic Health Evaluation 2, *SOFA* Sequential Organ Failure Assessment, *NUTRIC* Nutrition Risk in the Critically ill

^a^PaO2 / FiO2 > 200 indicates mild, PaO2 / FiO2 101–200 indicates moderate and PaO2 / FiO2 ≤ 100 indicates severe ARDS

## Discussion

There is a high prevalence of malnutrition among hospitalized patients; critically ill patients are also more vulnerable to this condition [26, 27]. Poor nutritional status can affect therapies’ effectiveness, duration, and severity of disease and also increases the mortality chance in COVID-19 patients [28–30]. Therefore, a significant focus should be placed on nutritional assessment in these individuals.

The Nutrition Risk Screening-2002 (NRS-2002) and NUTRIC are two main nutritional assessment tools to evaluate nutritional risk in critically ill patients [31]. The NRS-2002 is mainly based on traditional nutrition parameters such as weight loss and changes in food intake [32]. The NUTRIC score is based on the severity of the disease rather than traditional nutrition markers [33]. In a retrospective analysis in 2018, it was shown that when it comes to evaluating malnutrition risk in ICU patients, NUTRIC outperforms NRS-2002 [34]. The NUTRIC score has been shown to be useful in estimating outcomes in critically ill patients in numerous investigations [35–38]. In our study, we evaluated the effectiveness of the NUTRIC score in critically ill patients affected by COVID-19 admitted to the ICU in the Iranian population. To our knowledge, our study is the first work evaluating the effectiveness of the NUTRIC score in critically ill COVID-19 patients in Iran.

In our results, the mean age of deceased patients was higher than survived individuals, although the difference was not statistically significant between the two groups, many studies have considered older age as an obvious risk factor in COVID-19 that can increase the chance of death in these patients [39–42]. The duration of both hospital and ICU stays was longer in survived patients than in non-survivors. Higher severity of disease in deceased patients might be the reason of early death that consequently can shorten hospital duration.

In terms of CRP, our findings revealed that the mean CRP level was significantly higher in deceased patients than in survivors. A meta-analysis conducted in 2020 demonstrated that CRP levels were lower in COVID-19 patients with the non-severe disease [43]. There are also a number of studies that suggest that high CRP concentration increases mortality chance in COVID-19 patients [44–47]. Inflammation has a key role in COVID-19 disease. Virus infection activates immune responses that result in cytokine storm and systemic inflammation, thus, elevated levels of inflammatory markers will be observed in patients [48]. Moreover, bacterial co-infections in COVID-19 patients can increase CRP levels and result in adverse clinical consequences [49].

Based on our findings, PH was lower in deceased patients than in survivors and it was lower than the normal range. Low PH is a common problem in various diseases. In COVID-19, adjusting acid-base balance is a therapeutic strategy that can prevent the progression of the disease, as low PH can facilitate viral multiplication and increase inflammatory response [50, 51]. Furthermore, one study found that acidosis was more common among fatal individuals [42]. We found that deceased patients had higher PCO2 than patients who survived but it was still within the normal range.

About the mNUTRIC score, the mean score of the two groups of deceased and survived individuals were both lower than 5. However, the difference was statistically significant between two groups. Survived patients had a lower score than deceased individuals. Previous studies have demonstrated that NUTRIC can be used as a predictor of malnutrition risk and mortality in critically ill COVID-19 patients and a higher NUTRIC score was associated with a higher mortality rate [52–55].

In this study, we found that there was a positive relationship between NUTRIC score and age, CRP, AST, and LDH. It has previously been stated that poor nutrition status is associated with age and inflammation [56–60]. In addition, it is known that malnutrition can make changes in the levels of liver enzymes and increase AST and ALT because of its impact on liver cells [61–63]. High LDH levels have been shown to be a predictor of COVID-19 severity and mortality. Malnutrition can exacerbate the severity of the disease and as a result, LDH levels might increase [64, 65]. Based on our findings, the mNUTRIC score was inversely correlated with hospital duration, albumin, and PH. In a study, it is reported that poor nutritional condition was associated with low albumin levels [66]. Malnutrition can increase the mortality rate in COVID-19 patients [14], which may be one reason for lower hospital duration.

In the current study, a higher mNUTRIC score was associated with higher mortality chance, however, it was not statistically significant. Nevertheless, in some other studies, it is shown that a high NUTRIC score was related to an increased mortality rate in a smaller number of COVID-19 patients [54, 55, 67]. More studies should be done to find results that are statistically meaningful as well. Severe disease and PCO2 were also correlated with the chance of death. In previous studies, it has been shown that other factors like comorbidities, obesity, D-dimer, low SpO2/FiO2 ratio, male gender, and higher age are associated with disease severity and mortality [68–70].

Our study has some limitations that should be considered: 1) the sample size was relatively small. 2) There was a lack of more clinical tests. 3) Our design was not a multi-center study. 4) Following up was not possible.

## Conclusion

In conclusion, our findings demonstrated that a higher NUTRIC score was non-significantly correlated with higher mortality chance in patients with COVID-19. However, the higher PCO2 and increase in the severity of the disease were also significantly associated with higher odds of mortality. In addition, poor nutritional status and mortality were higher in patients with higher CRP levels and lower PH.

## Data Availability

The datasets which was generated and analyzed during the current study and used for the preparation of the manuscript are included in the article submitted for publication.
